# Common Noctule Bats Are Sexually Dimorphic in Migratory Behaviour and Body Size but Not Wing Shape

**DOI:** 10.1371/journal.pone.0167027

**Published:** 2016-11-23

**Authors:** M. Teague O’Mara, Karla Bauer, Dominik Blank, Justin W. Baldwin, Dina K. N. Dechmann

**Affiliations:** 1 Department of Migration and Immuno-ecology, Max Planck Institute for Ornithology, Radolfzell, Germany; 2 Department of Biology, University of Konstanz, Konstanz, Germany; 3 Smithsonian Tropical Research Institute, Balboa, Ancón, Panamá; Brown University, UNITED STATES

## Abstract

Within the large order of bats, sexual size dimorphism measured by forearm length and body mass is often female-biased. Several studies have explained this through the effects on load carrying during pregnancy, intrasexual competition, as well as the fecundity and thermoregulation advantages of increased female body size. We hypothesized that wing shape should differ along with size and be under variable selection pressure in a species where there are large differences in flight behaviour. We tested whether load carrying, sex differential migration, or reproductive advantages of large females affect size and wing shape dimorphism in the common noctule (*Nyctalus noctula*), in which females are typically larger than males and only females migrate long distances each year. We tested for univariate and multivariate size and shape dimorphism using data sets derived from wing photos and biometric data collected during pre-migratory spring captures in Switzerland. Females had forearms that are on average 1% longer than males and are 1% heavier than males after emerging from hibernation, but we found no sex differences in other size, shape, or other functional characters in any wing parameters during this pre-migratory period. Female-biased size dimorphism without wing shape differences indicates that reproductive advantages of big mothers are most likely responsible for sexual dimorphism in this species, not load compensation or shape differences favouring aerodynamic efficiency during pregnancy or migration. Despite large behavioural and ecological sex differences, morphology associated with a specialized feeding niche may limit potential dimorphism in narrow-winged bats such as common noctules and the dramatic differences in migratory behaviour may then be accomplished through plasticity in wing kinematics.

## Introduction

Among the large order of bats, sexual size dimorphism (SSD) is often female biased or absent [1–4] despite social structures that are typically linked to sexual selection and male-biased SSD in other groups of mammals, including harems and leks [5, 6]. Female-biased SSD in bats and most mammals is typically seen as a result of positive natural selection for larger females [5, 7, 8]. Large females may be more competitive or better or more efficient mothers [5, 9], and they may be able to thermoregulate more effectively due to a decreased surface to volume ratio [2, 10, 11]. These “big mothers” may then maximize gestational efficiency, invest more in offspring, produce higher quality milk, and may have better control of the timing of their reproduction [2, 10]. Big mothers may be particularly advantageous to species that migrate over long distances, as they time reproduction to maximum food availability so that both the females and their offspring can migrate back to over-wintering sites. This hypothesis is supported by studies that find longer forearms in females of most tested species of vespertilionids, however there are no other relationships to load carrying after adjusting for size [2]. When more detailed wing shape is examined, there does appear to be a relationship between sex dimorphism and load carrying in other bat families [4, 12]. The relationship between female-biased size differences, selection for big mothers, and other morphological sex dimorphism in load carrying and shape is, therefore, still unresolved.

Sex differences may not be only limited to size but also to shape. Wing morphology is correlated with flight behaviour and is particularly sensitive to variation in ecology [13–15]. Small differences in size and/or wing loading can have large effects on manoeuvrability [16, 17] and subsequently on feeding efficiency and habitat segregation. For example, animals that hunt in open space generally show high wing loading (wings that generate relatively large force) and high aspect ratio (wings that are relatively long) that result in high maximum speeds but low manoeuvrability at slow speeds [13, 14]. In contrast, animals that forage from an observational perch or through cluttered habitat show broad wings with low aspect ratio that allow for high manoeuvrability at slow foraging speeds but are less efficient for high speeds or long flights. Long-distance migration may have effects on wing shape that differ from the effects of foraging mode, and sex differences in migration behaviour may be reflected in wing shape. Migrants may use long and pointed wings with high aspect ratio and high wing loading [13, 18] to fly farther, faster and with less resistance as shown by variation among [19] and within species [20, 21], but this may then restrict them to open-aerial foraging niches. Alternatively, a more generalized wing shape with shorter and more rounded wing tips may force species to use longer stopovers along a migration route to compensate for decreased flight efficiency [22]. This relationship between wing shape, energetic expenditure, and ecology, implies that wing size and shape should be particularly sensitive to sex differences in behaviour and physiology.

Female load carrying and flight efficiency may directly impact wing shape dimorphism with opposing optimal shapes. Female bats give birth to offspring with a total litter mass of nearly 50% of the mother’s own mass [13, 23] and then often transport pups between roosting sites until they are up to 70% of adult mass. Litter mass is constrained by the aerodynamics of flight and load carrying, selecting for lower aspect ratio and low wing loading rather than increased body size [1, 24] and several studies have linked this to the increased load carried by females when pregnant and lactating [1, 2, 4, 12, 24]. Likewise, sex-biased migration may be a driver of differences in wing morphology since wing shape variation within a species directly affects their flight performance and energy use [20, 21], and higher aspect ratio and more pointed wing shapes are more efficient. In the best-studied European long-distance migrant, the common noctule (*Nyctalus noctula*), females move north or northeast each spring to raise offspring in their natal maternity colonies [25, 26]. After young have fledged, females and their offspring migrate back to over-wintering habitats to mate and prepare for hibernation. While both sexes migrate south at least once in their life, females will migrate to maternity colonies each year [3] and males remain in wintering areas or only move smaller distances [27]. Female noctules are larger than males in both body mass and forearm lengths (a standard size measure in bats) [3], and may therefore be under stronger pressure to optimize wing shape for repeated long-distance migrations, as arriving at maternity colonies with high body condition would be particularly advantageous.

Sex differences in body size and the size and shape of wings in common noctules may then be a response to three non-exclusive pressures. We predict that H1) if “big mothers” are better mothers, particularly because of thermoregulatory limitations during pregnancy and lactation, females should be larger overall, without relatively larger wings [2], or H2) if wing shape dimorphism is an adaptation to load carrying, relative wing surface area of females should be larger [1] particularly at the tips [12], or H3) if wing dimorphism was mainly driven by sex dimorphic migration, females should have higher aspect ratio and more pointed wingtips leading to lower cost of transport [13, 19, 21]. We used traditional univariate measures collected from live common noctules, as well as two-dimensional geometric morphometrics from wing photos to test our hypotheses.

## Methods

### Captures and wing photos

We collected data from common noctules (*Nyctalus noctula*) sampled from three bat box populations in Switzerland (Seeburgpark Kreuzlingen: 47.649928°, 9.186123°; Allmend Frauenfeld: 47.580268°, 8.906434°; Bischofszell: 47.485279°, 9.218046°) over five years (2012–2016). Boxes were checked up to three times between April and May and measurements were taken from all bats.

Captured bats were placed individually in soft cloth bags until processing. The mass of each bat was measured (± 0.5 g; Pesola spring balance), the forearm measured with calipers (± 0.1 mm), and the sex and reproductive status determined (females: nulliparous/post-lactating; males: scrotal (reproductively active) or non-reproductive as assessed by the filling of the epididymes). We released bats back in their roost box after all data were collected. Body condition was calculated as mass divided by forearm length [28, 29]. We use forearm length as our measured indicator of size as this is the most commonly collected measure of bats. It is measured across a single bone (with exception of included carpals), and does not fluctuate once individuals have reached adult size. Body mass in bats, however, fluctuates dramatically based on the time of year, and the time of day. While our bats were all sampled from their roost boxes in the spring, there is variation across years and the time since emerging from hibernation that can affect mass changes [3, 27, 30]. Each bat was marked with a subcutaneous PIT-tag (ID100; Euro ID, Weilerswist, Germany), injected under the dorsal skin. No bat was sampled more than once, which resulted in a total 445 individuals (F: 357, M: 88). We analysed the data using t-tests to identify the effect of sex on forearm length, mass, and body condition.[31]

### Ethics Statement

All handling and sampling of the bats in Switzerland was approved by the Veterinäramt Thurgau (FIBL1/12). All methods conformed to the ASAB/ABS Guidelines for the Use of Animals in Research.

### Multivariate wing shape

**Fig 1:**
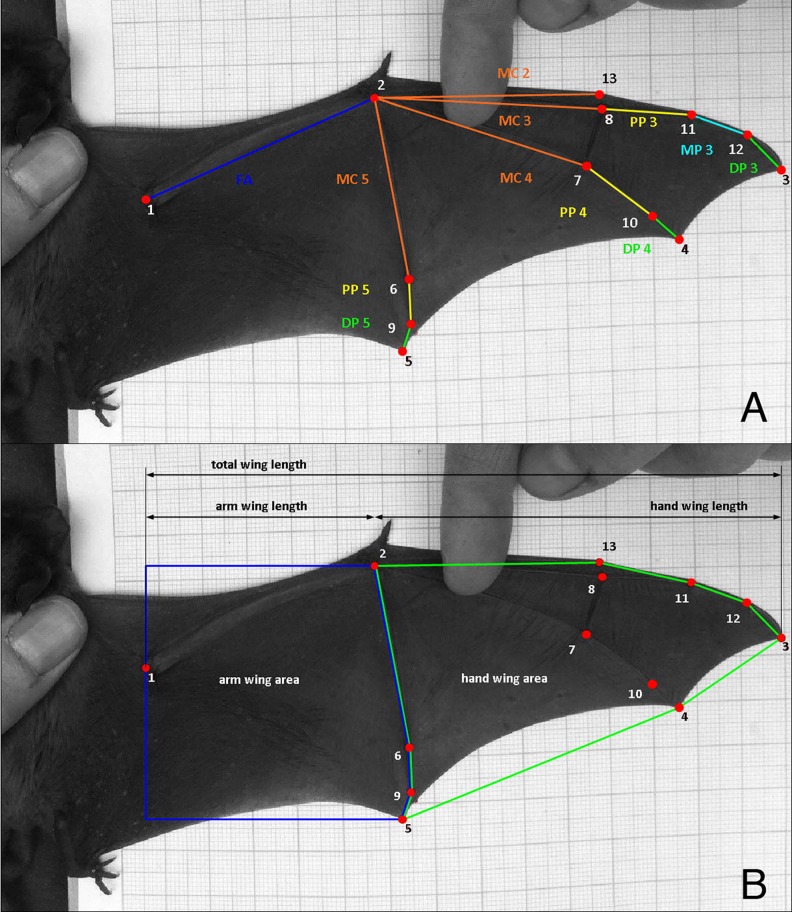
Wing morphology of *Nyctalus noctula*. A) Right wing of *N*. *noctula* with 13 landmarks that were used to calculate individual bone lengths. The blue line represents the forearm length, the metacarpals are shown in orange, the proximal phalanges in yellow, the medial phalanx of digit 3 in cyan, and the distal phalanges are in green. B) Right wing of *Nyctalus noctula* showing the calculation of areas, and linear distances.

In a subset of the captures, we took a picture of the wing on graph paper mounted on a clipboard by gently stretching the right wing until the elbow was fully extended (Fig 1). From the resulting 135 wing photos (94 female, 41 male) we were able to use different subsets in the various analyses (e.g. an image of a wing that was not stretched in the ideal position might allow analysis of landmarks and bones, but not all areas; see results). Only the best photo was used per individual and wherever possible we placed 13 landmarks on each wing photo (Fig 1) using ImageJ (Rasband, 1997–2012) and the Pointpicker Java extension. Each landmark was placed on a morphologically relevant point: 1: elbow, 2: wrist, 3: distal tip of third finger, 4: distal tip of fourth finger, 5: distal tip of fifth finger, 6: metacarpal-phalangeal joint on fifth finger, 7: metacarpal-phalangeal joint on fourth finger, 8: metacarpal-phalangeal joint on third finger, 9: interphalangeal joint between phalanges 1 and 2 on fifth finger, 10: interphalangeal joint between phalanges 1 and 2 on fourth finger, 11: interphalangeal joint between phalanges 1 and 2 on the third finger, 12: interphalangeal joint between phalanges 2 and 3 on the third finger, 13: metacarpal-phalangeal joint on second finger. After scaling size using the graph paper, we calculated individual bone lengths using Euclidean distances between the relevant landmarks (Fig 1A) and the following traits based on straight-line distances between associated landmarks (Fig 1A): forearm length (1–2), wing length (perpendicular distance between points 1–3), arm wing length (perpendicular distance between 1–2), and hand wing length (perpendicular distance between 2–3). We further divided the wing area into arm wing (plagiopatagium: area up to the fifth phalanx) and the hand wing (dactylopatagium: area from the fifth phalanx to the wing tip). We note that taking this approach removes some of the area closest to the body, but our standardized method ensures that consistent areas are measured for each portion of the wing. We calculated wing areas (Fig 1B) by defining two bordering polygons that can be consistently defined and are not subject to positioning of the foot or estimating the location of the shoulder [12]. The proximal margin of the hand wing polygon was defined by a vertical line through the elbow, and the cranial and caudal margins by perpendicular lines from the wrist and end of the fifth digit, respectively. We did not correct for any potential distortion in the photos, but positioned the wing in the centre of the photo to minimize distortion due to lens curvature. Furthermore, non-systematic distortion in the photos would be more likely to mask differences than suggest false positives, making rejection of our null hypotheses more difficult. To measure our marker placement error, we re-sampled a subset of 10 individuals and re-placed the markers a second time [32]. We then used a MANOVA to estimate any difference between the two sets of points and found no statistical difference among the landmark sets (all p-values > 0.99). There was a mean difference in Euclidean distance of 0.03 ± 0.64% among points and a maximum Euclidean distance between points was 3.07 pixels. All wing photo measurements were carried out by DB.

For each individual wing we then calculated morphological traits that are relevant to long-distance migration and load carrying [13]. These include wing aspect ratio (wing length^2^ / wing area), wing loading (mass*g / wing area), tip length ratio (hand wing length / arm wing length), tip area ratio (hand wing area / arm wing area), and tip shape index (tip area ratio / tip length ratio–tip area ratio). We evaluated sex differences in size and shape measures at α = 0.05 using t-tests in R 3.3.1 [33].

We also used a geometric morphometric approach to assess if male and female wing shapes differ across the wing as some shape differences can be obscured by traditional indices [12, 34, 35]. We used a subset of the wing photos where all 13 landmarks were visible (82 females, 27 males). Landmarks were then rotated, translated, and scaled using generalized Procrustes analysis to remove the effects of size and other non-shape parameters [36, 37] in MorphoJ [38]. We used a t-test to examine sex differences in centroid size and a Principal Component Analysis (PCA) of Procrustes coordinates to identify individual measures that influence differences in wing shape between sexes. We further tested the effects of sex and centroid size on shape using a MANOVA. The full dataset is included as Supporting Information (S1 Data).

## Results

### Captures

**Fig 2:**
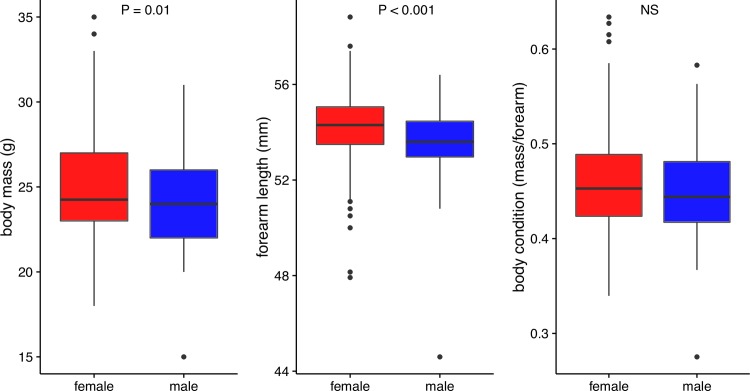
**Boxplots of forearm lengths (A), body mass (B), and body condition (C) of female (red) and male (blue) noctules.** Females have longer forearms and higher body mass.

We found sex differences in forearm length and body mass between female (N = 357) and male (N = 88) common noctules at the time of capture. Females had forearms that were 1% longer than males in mean length (F: mean ± SD: 54.2 ± 1.4, range: 50.0–58.2; M: 53.5 ± 1.5, range: 44.6–56.4; t_125.23_ = 3.96, *P* < 0.001 Fig 2; Table 1). Females were 1% heavier than males (F: 24.95 ± 2.68, range: 18–35 g; M: 24.12 ± 2.72, range: 15–32 g; t_132.05_ = 2.55, *P* = 0.01, Fig 2; Table 1), but there were no sex differences in overall body condition (F: 0.46 ± 0.04, M: 0.45 ± 0.05; t_126.11_ = 1.66, *P* = 0.10, Fig 2; Table 1).

**Table 1:** Summary statistics for noctule wing parameters. Mean (± sd) for sex differences in wing shape and size variables collected from wing photos of 135 common noctules (94 female, 41 male).

	Female	Male	t-statistic	df	p-value
Mass (g)	24.95 ± 2.68	24.12 ± 2.72	2.55	132.05	0.01
Forearm length (mm)	54.2 ± 1.4	53.5 ± 1.5	3.96	125.23	< 0.001
Body condition (mass / forearm)	0.46 ± 0.05	0.45 ± 0.05	1.66	126.11	0.10
Centroid size	1436.72 ± 508.93	1650.10 ± 475.87	-1.99	47.13	0.06
Digit 3 (mm)	94.05 ± 4.71	94.03 ± 8.24	0.01	31.69	0.99
Digit 4 (mm)	76.1 ± 3.6	75.59 ± 3.17	0.82	86.36	0.41
Digit 5 (mm)	57.62 ± 2.66	57.21 ± 2.19	0.92	88.73	0.36
Metacarpal 2 (mm)	49.77 ± 2.44	49.57 ± 5.41	0.21	39.56	0.83
Metacarpal 3 (mm)	50.39 ± 2.27	49.67 ± 1.81	1.84	74.75	0.07
Proximal phalanx 3 (mm)	19.49 ± 1.6	19.05 ± 1.56	1.25	45.25	0.22
Medial phalanx 3 (mm)	13.51 ± 1.23	13.7 ± 1.88	-0.55	42.54	0.59
Distal phalanx 3 (mm)	10.59 ± 0.73	11.37 ± 5.2	-0.91	37.61	0.37
Metacarpal 4 (mm)	49.46 ± 2.25	49.14 ± 1.89	0.86	89.81	0.39
Proximal phalanx 4 (mm)	18.65 ± 1.21	18.5 ± 1.11	0.69	82.88	0.49
Distal phalanx 4 (mm)	7.94 ± 0.86	7.95 ± 0.78	-0.11	84.03	0.91
Metacarpal 5 (mm)	41.12 ± 1.84	40.71 ± 1.57	1.33	88.36	0.19
Proximal phalanx 5 (mm)	10.26 ± 0.7	10.21 ± 0.77	0.33	70.33	0.74
Distal phalanx 5 (mm)	6.24 ± 0.56	6.2 ± 0.59	0.31	70.4	0.76
Wing length (mm)	141.18 ± 6.29	139.5 ± 4.88	1.67	93.99	0.10
Hand wing length (mm)	55.79 ± 2.98	55.29 ± 2.65	0.97	82.28	0.34
Arm wing length (mm)	85.39 ± 4.46	84.21 ± 3.67	1.6	88.88	0.11
Wing area (mm^2^)	6200.17 ± 524.26	6044.98 ± 406.2	1.6	56.85	0.12
Arm wing area (mm^2^)	3157.68 ± 295.92	3111.38 ± 271.71	0.87	76.99	0.39
Hand wing area (mm^2^)	3053.34 ± 275.47	2985.45 ± 233.66	1.25	51.76	0.22
Propatagium area (mm^2^)	520.21 ± 147.72	519.35 ± 146.55	0.03	74.2	0.98
Plagiopatagium area (mm^2^)	2637.47 ± 347.25	2588.91 ± 307.36	0.8	79.76	0.43
Dactylopatagium minus area (mm^2^)	92.24 ± 18.78	96.46 ± 20.79	-0.94	40.72	0.35
Dactylopatagium medius area (mm^2^)	1052.92 ± 112.74	1011.78 ± 109.8	1.68	45.43	0.10
Dactylopatagium major area (mm^2^)	1902.06 ± 158.63	1891.65 ± 143.48	0.37	81.26	0.71
Aspect ratio	6.45 ± 0.24	6.38 ± 0.21	1.36	49.04	0.18
Wing loading (Nm^-2^)	20.02 ± 3.23	19.64 ± 1.96	0.72	74.79	0.47
Tip length ratio	0.65 ± 0.04	0.66 ± 0.04	-0.44	73.24	0.66
Tip area ratio	0.97 ± 0.08	0.98 ± 0.09	-0.34	38.98	0.73
Tip shape index	3.35 ± 1.22	3.5 ± 1.71	-0.4	35.11	0.69

### Multivariate wing measures

We analysed wing photos of 94 female bats and 41 males where we could calculate wing areas and bone lengths. Forearm lengths measured from photos were 0.34 mm shorter than forearm lengths taken externally with calipers during captures (*F*_1,132_ = 47.63, *P* < 0.001), likely as it was difficult to locate the olecranon process on the photos that serves as the proximal landmark of the elbow. For individuals where both methods were used, our measurements were in agreement with a moderate degree of correlation between the two measures (Pearson correlation coefficient *r* = 0.51). Measurements taken from the photos were consistent with field measures showing females with longer forearm lengths (*t*_125.23_ = 3.96, *P* < 0.001, Table 1). However, no other measures taken from these 2D points revealed any sex differences in size or shape (Table 1).

**Fig 3:**
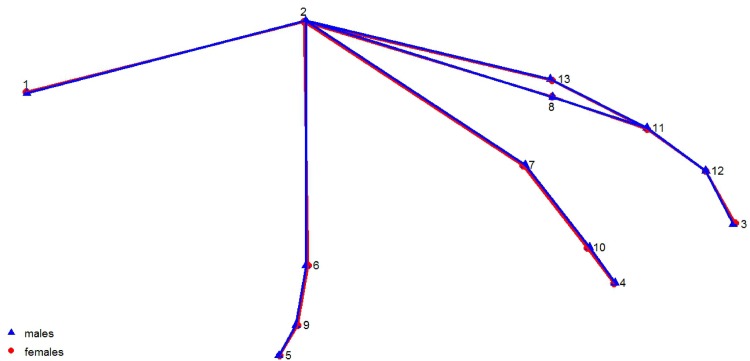
Wireframe shape distortions based on the first principal component in the geometric morphometric analysis. No sex differences in wing shape were found.

**Fig 4:**
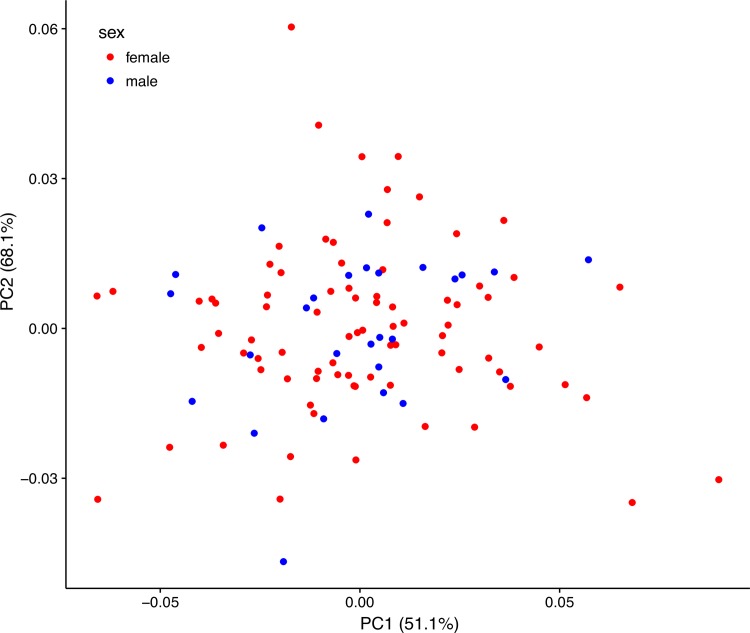
Principal component 1 vs 2 of Procrustes scaled coordinates in the geometric morphometric analysis. The first two principal components account for a cumulative 51.1% and 68.1% of the variation, respectively.

A further subset (82 female, 27 male) of the 135 wing photos above had all 13 anatomical points visible for analysis. A PCA on the covariance matrix generated from Procrustes-fitted landmark data revealed that there were no sex differences in the shape of noctule wings and showed nearly complete overlap in shape space, with PC1 and PC2 (Figs 3 & 4) accounting for 68.1% of the cumulative variation in the data set. There was no influence of centroid size, sex, or their interaction on wing shape in the first three principal components (PC1: *F*_3, 105_ = 1.684, p = 0.18, PC2: *F*_3, 105_ = 1.152, *P* = 0.33; PC3: *F*_3, 105_ = 0.898, *P* = 0.45). Furthermore a MANOVA of the Procrustes coordinates showed a trend towards sex differences in centroid size (F_1, 107_ = 3.68, *P =* 0.06), with slightly larger male centroids, and in the size-corrected shape space (F_22, 2354_ = 1.14, *P =* 0.08). We therefore ran a final discriminant function analysis using 1000 permutations in MorphoJ and found that we were not able to reliably classify sex by shape of the wing alone (*P* = 0.38).

## Discussion

Despite sexual size dimorphism in the forearm length and body mass of the common noctule, and strong sex dimorphism in seasonal load carrying due to pregnancy and migration [3], there were no sex differences in any of the wing shape and size parameters that we examined. The lack of shape and size dimorphism in the wings of common noctules leads us to conclude that selection on larger females is likely responsible for the small but statistically significant size and mass differences in common noctules.

Forearm length is the primary size variable measured in live bats, with differences between females and males typically 1–2% [2]. Forearm size is measured across a single bone (the radius) with minor inclusions of the carpals and ulna, making this measure both easily collected and consistent both within and among species. While forearm size may not be the best measure of size among species for both functional and phylogenetic reasons, it performs well as an intraspecific size measure [13]. In our common noctules, forearm lengths we measured were similar to those in other populations of common noctules (overall mean F: 54.7 ± 1.2, range: 49.8–57.5 mm; M: 53.6 ± 1.4, range: 48.0–56.5 mm, [3]), and females and males in our sample differed by 0.9 mm. The length of the fifth finger (ca 57 mm for both sexes in our study) was also similar to previous results (F: 56.4 ± 1.3, range 53.7–59.0; M: 55.5 ± 1.6. range 52.0–58.9, [3]). But as we now show, this overall difference of about 1.0 mm does not result in any overall sex differences in wing size or shape in the common noctule. In more broad-winged bat species, shape differences vary by species. Females of the vespertilionid bat *Nycticeius humeralis* have longer forearms, but there are no sex differences in wing loading [39], while female forearms are longer in another vespertilionid, *Myotis myotis*, where females also have longer third and fifth fingers [40], but it is unknown how overall shape and wing area are affected. Longer female forearm lengths in the phyllostomid bat *Artibeus lituratus* are accompanied by larger overall wings [4], but in the closely related *Sturnira lilium*, there are no sex differences in forearm length or overall wing area [12]. Female *S*. *lilium* do show larger wing areas near their wing tips, likely as an adaptation to decrease loading and increase lift potential while carrying heavy offspring [12]. These few multivariate analyses of bat wing shape show a broad diversity, but also highlight that sexual dimorphism in a common size measure for bats may have mixed functional relevance to dimorphism in other aspects of the wing.

While our data are consistent with the hypothesis that sexual size dimorphism is a result of natural selection for big mothers, it is not clear how these relatively small differences in female size relative to males affects reproductive success or fecundity [2, 5]. There is a large effect of migration status on sex differences in body condition [27]. Once females have started spring migration they are in better body condition than males [27]. This rapid weight gain prior to long-distance migration to natal maternity colonies [26] may be essential to successful and early reproduction at maternity colonies as females may increase body mass by up to 50% during pregnancy [41]. However, it is unknown if larger noctule mothers give birth to larger offspring, are able to bring offspring to term earlier, or are more successful at raising these infants.

Female bats may increase their mass by 50% during pregnancy and up to 70% when carrying non-volant offspring between roosts [42], but this increased load does not impact noctule wing shape. Since both female and male body mass and body condition undergo large fluctuations across the year there may be equivalent pressure on the wing surfaces of both sexes. Studies of broad-winged phyllostomid bats have found females with larger absolute and relative wing sizes [4]. There is some evidence that this size and shape difference in the wing is limited to the dactylopatagium, despite a lack of sex differences in the length of the underlying bone structure, including the forearm [12]. Both of these studies implicate the need for relatively larger wing areas for females to maintain manoeuvrability during pregnancy, and lactation as they carry their offspring until they reach 70% of adult body size. However, in bats with long and narrow wings the potential to modify the shape or size of wings may be tightly constrained by aerodynamic performance in a relatively narrow niche.

Noctules have some of the highest wing aspect ratio and wing loadings measured for bats [13]. Common noctules hunt for ephemeral insects in open air for 40–50 minutes per night [27], during which they may consume up to 30% of their body mass in insects (Dechmann & O’Mara, unpublished data). Our capture data of fasted pre-emergence individuals also show that they gain eight grams of fat (ca. 30% of body mass) over a 14 day period in the spring prior to migrating. Both radio telemetry and stable isotope studies of noctules suggest that females and males occupy overlapping niches in our study area [27], despite marked niche partitioning among females of varying reproductive states elsewhere [43]. Since strong directional selection on traits can limit modularity and tightly integrate the characters comprising a trait [44, 45], there may be less flexibility to modify noctule wing shapes. The integration of wing components necessary to navigate the aerial insectivore niche may limit the potential for morphological variation between females and males, but allow sufficient flexibility for sex differences in foraging behaviour to evolve [46]. In contrast, the high aspect ratios and low wing loading of phyllostomid bats may be more permissive of changes in overall wing shape and subsequent sexual dimorphism [4, 12].

The lack of sex differences in our measures of 2D wing shape of outstretched wings does not necessarily preclude functional differences between female and male flight. Bat wings are complex structures and the many joints, elastic wing membrane, and intervening musculature within the membrane give bats enormous kinematic flexibility that is not captured in 2D structure. High-speed video demonstrates different scaling relationships of body size to wing size than other methods, with bats in flight revealing larger exponential body size–wing area relationships than bats that are measured in the flatter posture used in our study, and large bats will reduce wing loading by using different wing postures than smaller bats [47]. Interestingly, the only migratory species in that study (*Eidolon helvum*) shows more variation in wing camber than the non-migratory flying foxes [47]. Furthermore, bats with very different wing shapes can show similar kinematics when flying at higher speeds [48]. When the wingbeat kinematics of similar-sized broad-winged and narrow-winged bats are compared, it seems that the largest difference in flight efficiency is at slow speeds [30, 48]. Narrow-winged bats may have to make larger adjustments to their wingbeats when flying at slow speeds than do bats with broader wings. There may then be stronger selection on flight efficiency at low vs. high speeds, but it is yet unclear how efficiency differs at the high speeds estimated for migratory noctules [27].

In summary, despite dramatic differences in behaviour and the potential for energetic pressures, there are no sex differences in the wing size or shape of common noctules. Our results suggest that outside of the apparent widespread nature of female-biased sexual dimorphism in bats, there may be more ecological and niche limitations on wing shape dimorphism than previously appreciated. While female noctules must migrate long distances and carry large loads they may be able to modulate wing kinematics sufficiently to compensate for these very different behavioural states. Careful examination of parameters across the entire wing in addition to forearm length will be necessary to fully evaluate the relationship of sexual dimorphism to bat behaviour and evolution.

## Supporting Information

S1 Data Capture data, measurements, and 2D point placement (X,Y) for *Nyctalus noctula*. Not all individuals had all aspects of data recorded for them. NA indicates no data collected for that variable. The 65 variables are: Bat PIT tag identity; bat mass; bat sex; measured forearm length; body condition; bat photo identity; coordinates scaled; X1; Y1; X2; Y2; X3; Y3; X4; Y4; X5; Y5; X6; Y6; X7; Y7; X8; Y8; X9; Y9; X10; Y10; X11; Y11; X12; Y12; X13; Y13; forearm length measured from photos; length of metacarpal 2; length of metacarpal 3; length of proximal phalanx 3; length of medial phalanx 3; length of distal phalanx 3; length of digit 3; length of metacarpal 4; length of proximal phalanx 4; length of distal phalanx 4; length of digit 4; length of metacarpal 5; length of proximal phalanx 5; length of distal phalanx 5; length of digit 5; wing length; wing area; arm wing length; hand wing length; arm wing area; hand wing area; propatagium area; plagiopatagium area; dactylopatagium minus area; dactylopatagium medius area; dactylopatagium major area; aspect ratio; wing loading; tip length ratio wing; tip area; tip shape index.
(TXT)
